# Sequential Perforator-Based Reconstruction of a Recurrent Stage IV Sacral Pressure Ulcer in a Paraplegic Patient: Primary Superior Gluteal Artery Perforator Flap Followed by a Freestyle Perforator Flap Salvage

**DOI:** 10.7759/cureus.107245

**Published:** 2026-04-17

**Authors:** Nay Aung Zin, Thura Kyaw, Aung Chan Thar, Shaoe Mein Hlyan Maung, Thinn Thiri Soe

**Affiliations:** 1 Orthopaedics and Traumatology, Kulhudhuffushi Regional Hospital, Kulhudhuffushi, MDV; 2 Orthopaedics and Trauma, Mandalay Orthopaedic Hospital, Mandalay, MMR; 3 General Surgery, Kulhudhuffushi Regional Hospital, Kulhudhuffushi, MDV; 4 Orthopaedic Surgery, Thulusdhoo Health Center, Male, MDV; 5 Emergency, Kulhudhuffushi Regional Hospital, Kulhudhuffushi, MDV

**Keywords:** freestyle perforator flap, pressure ulcer, reconstructive surgery, sacral ulcer, sgap flap, spinal cord injury

## Abstract

Stage IV sacral pressure injuries remain a major reconstructive challenge in patients with spinal cord injury due to persistent mechanical loading, impaired sensibility, and high recurrence rates. Perforator-based reconstruction has emerged as a preferred surgical strategy because it provides durable coverage while preserving muscle and maintaining future reconstructive options. We report the case of a 43-year-old man with T11-T12 spinal cord injury and paraplegia who presented with a stage IV sacral pressure ulcer measuring 11 × 9 cm with exposed sacral bone. Serial surgical debridement, including burring of exposed bone, reduced the wound size to 8 × 8 cm. There was no evidence of osteomyelitis, wound cultures were negative, and hematologic and nutritional parameters were optimized prior to reconstruction. The defect was reconstructed using a superior gluteal artery perforator (SGAP) flap measuring 8 × 8.5 cm, identified through intraoperative perforator exploration. Initial postoperative healing was satisfactory. However, four months later, recurrent ulceration developed at the sacral region. Following repeat debridement, salvage reconstruction was performed using a freestyle perforator flap. The wound healed completely, and the patient remained ulcer-free after six months of follow-up. This case demonstrates the value of perforator-preserving reconstructive strategies that enable secondary reconstruction for recurrent sacral pressure ulcers in patients with spinal cord injuries.

## Introduction

Pressure ulcers are a common complication among patients with spinal cord injury and prolonged immobility. The sacral region is particularly vulnerable due to continuous pressure exposure during supine positioning, impaired protective sensation, and shear forces [1]. Sacral pressure ulcers affect approximately 20-40% of patients with spinal cord injury. Stage IV pressure ulcers involve full-thickness soft tissue loss, exposing muscle, fascia, or bone, and often require surgical reconstruction for definitive management. Perforator flaps are skin and subcutaneous tissue flaps supplied by small vessels that perforate the underlying muscle, thereby preserving muscle function. Freestyle perforator flaps refer to flaps designed intraoperatively based on any suitable perforator identified during surgery [2].

Traditional surgical management frequently relied on musculocutaneous flaps, particularly the gluteus maximus flap. However, these techniques sacrifice functional muscle tissue and may limit reconstructive options in the event of recurrence [3]. With advances in perforator flap surgery, fasciocutaneous perforator flaps have become increasingly preferred due to their ability to preserve underlying muscle while providing reliable vascularized coverage [4].

The superior gluteal artery perforator (SGAP) flap was first described as a muscle-sparing alternative for sacral pressure ulcer reconstruction. This flap offers reliable perfusion, adequate arc of rotation, and minimal donor site morbidity [5]. Despite successful reconstruction, recurrence remains a well-recognized problem in patients with spinal cord injury due to persistent risk factors such as pressure loading, immobility, and impaired sensation [6]. Freestyle perforator flaps have further expanded reconstructive options by allowing surgeons to design flaps based on perforators identified intraoperatively, enabling tailored reconstruction even in previously operated fields [7].

We present a case of recurrent stage IV sacral pressure ulcer in a paraplegic patient managed with sequential perforator-based reconstruction using an SGAP flap followed by a freestyle perforator flap salvage procedure.

## Case presentation

A 43-year-old male patient with traumatic T11-T12 spinal cord injury and paraplegia presented with a chronic sacral pressure ulcer. The patient had been wheelchair-bound for approximately one year and developed progressive ulceration over the sacral region secondary to prolonged pressure and impaired sensation. The ulcer developed progressively over the year before presentation.

**Figure 1:**
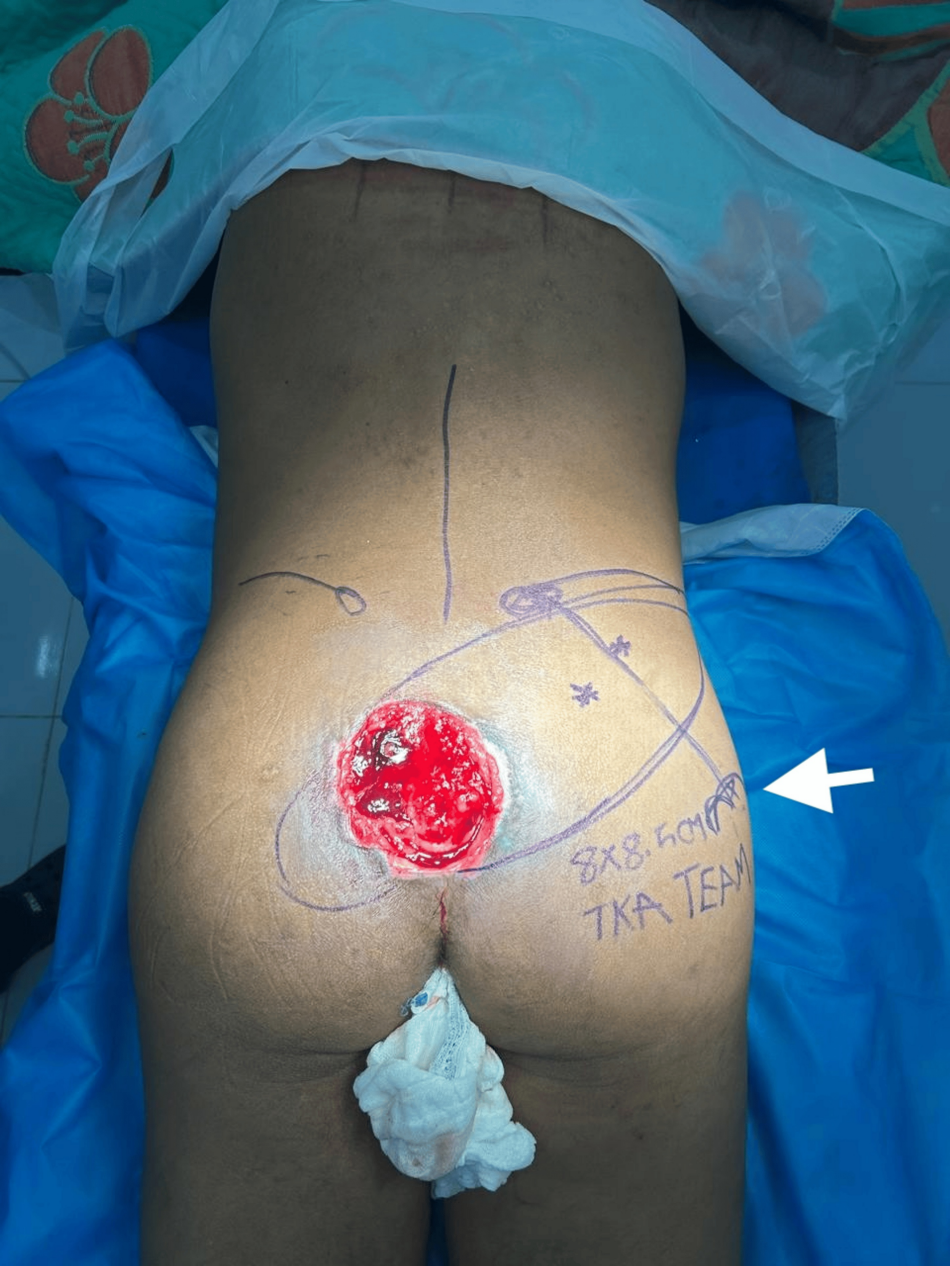
Preoperative sacral pressure ulcer and flap planning Preoperative photograph demonstrating a stage IV sacral pressure ulcer measuring approximately 11 × 9 cm with exposed sacral bone. Preoperative markings for a superior gluteal artery perforator (SGAP) flap are drawn on the right gluteal region. The arrow indicates the planned perforator location identified through intraoperative exploration.

Examination revealed a stage IV sacral pressure ulcer measuring approximately 11 × 9 cm with exposed sacral bone (Figure 1).

Laboratory investigations demonstrated normal inflammatory markers with no leukocytosis. Hemoglobin and serum albumin levels were within normal limits. Wound culture and sensitivity testing showed no bacterial growth, and there was no clinical or radiologic evidence of osteomyelitis.

Serial debridement and wound bed preparation were performed to remove necrotic tissue and establish a viable wound bed. During debridement, the exposed sacral bone was burred to eliminate potential bacterial colonization and promote bleeding cancellous bone. Then, the wound dimensions were reduced to approximately 8 × 8 cm, with healthy granulation tissue present.

**Figure 2:**
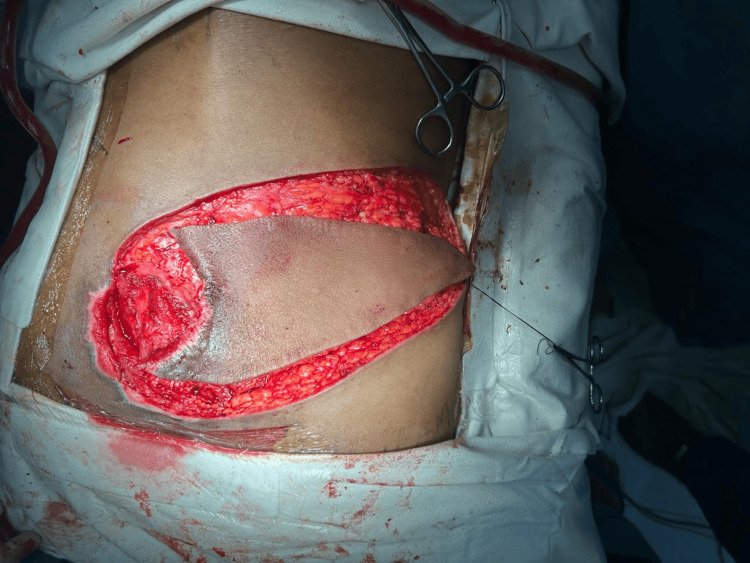
Flap design and elevation Intraoperative view showing elevation of a fasciocutaneous flap based on a superior gluteal artery perforator. The flap was designed to measure 8 × 8.5 cm to adequately cover the sacral defect after serial debridement.

Primary reconstruction was performed using a SGAP flap. Preoperative markings for the flap were made over the gluteal region (Figure 1). A fasciocutaneous flap measuring 8 × 8.5 cm was designed and elevated (Figure 2).

**Figure 3:**
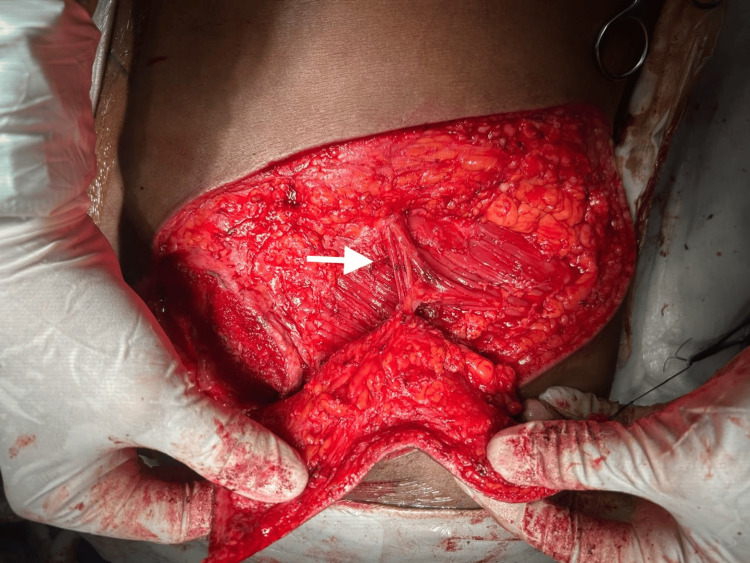
Identification of perforator vessels Intraoperative exposure demonstrating the perforator vessel arising from the gluteal region supplying the flap. Careful dissection was performed to preserve the perforator pedicle (arrow).

During surgery, perforator localization was achieved through intraoperative exploration, and a suitable perforator arising from the superior gluteal artery was identified (Figure 3).

**Figure 4:**
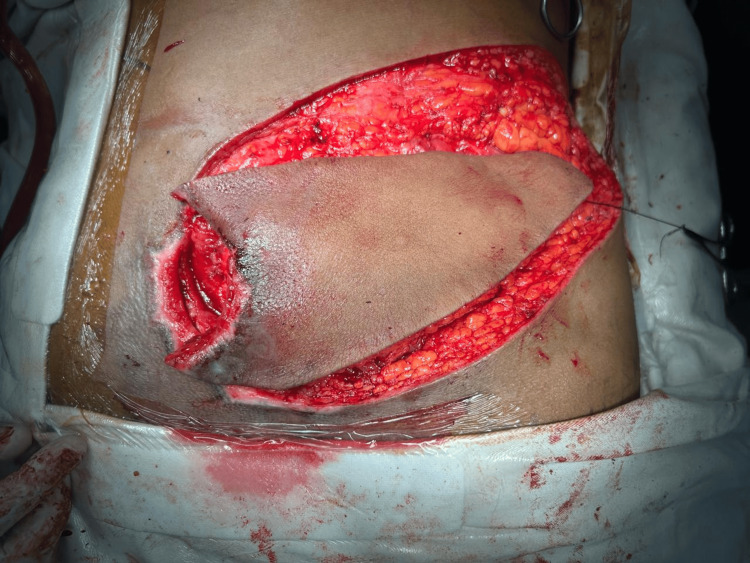
Flap transposition The superior gluteal artery perforator (SGAP) flap is mobilized and rotated toward the sacral defect. The wound bed shows healthy granulation tissue after serial debridement and burring of the exposed sacral bone.

The flap was carefully dissected while preserving the perforator pedicle and then rotated toward the sacral defect (Figure 4).

**Figure 5:**
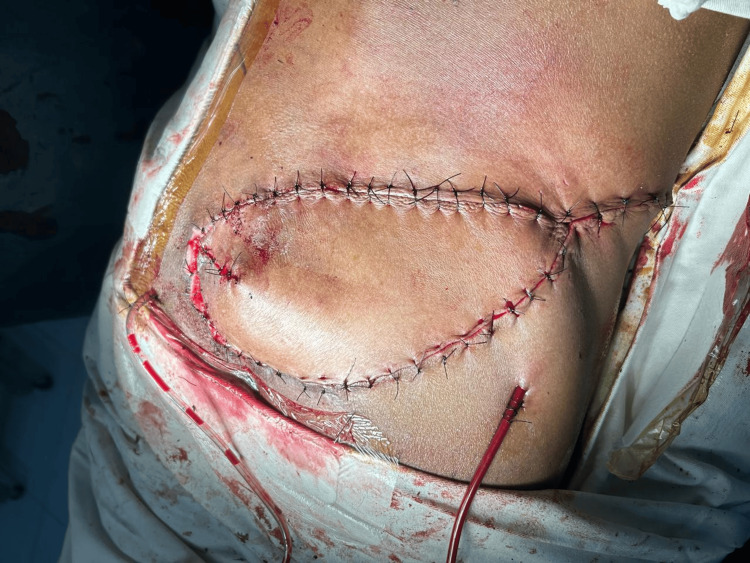
Flap inset Completion of flap inset and donor site closure with placement of a closed suction drainage.

Following adequate mobilization, the flap was inset into the defect to achieve complete coverage. The donor site was closed primarily, and a closed suction drain was placed beneath the flap. The immediate postoperative period was uneventful, and the flap demonstrated good viability (Figure 5).

**Figure 6:**
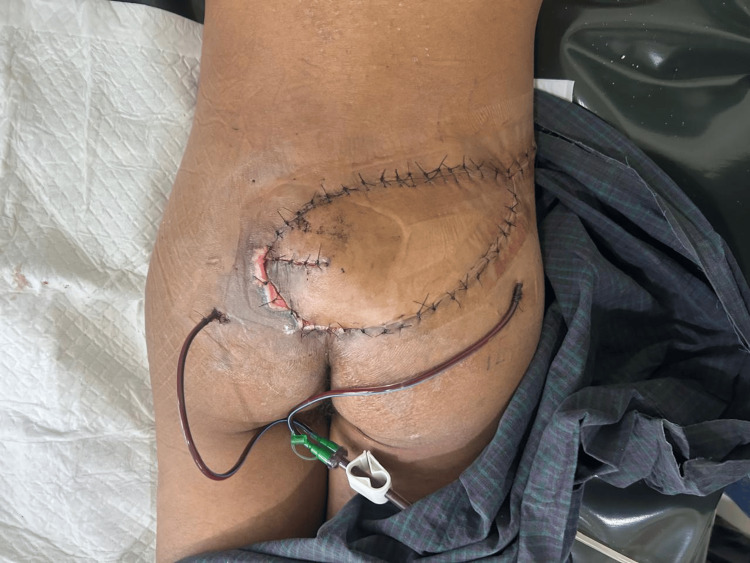
Early postoperative appearance Postoperative view showing viable flap coverage of the sacral defect with intact sutures and surgical drain in situ.

**Figure 7:**
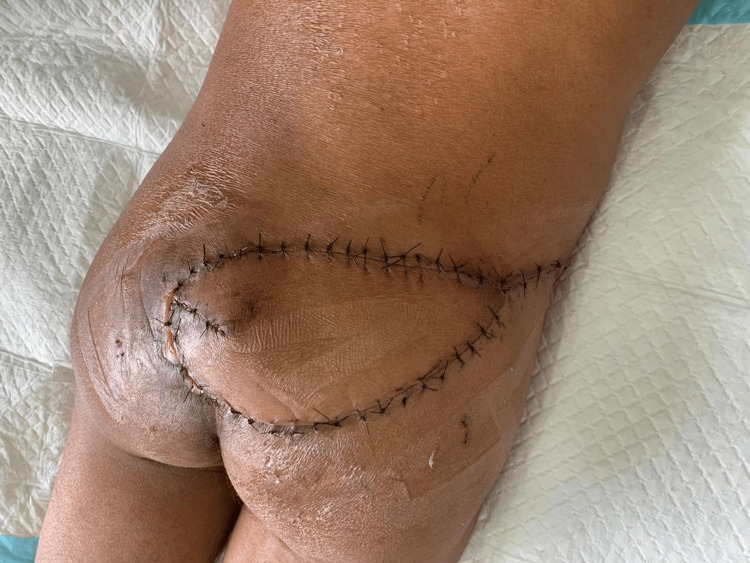
Two-week postoperative follow-up Healing flap with satisfactory tissue coverage and viable skin paddle. Sutures remain in place with no evidence of infection or necrosis.

At the early postoperative follow-up, the surgical site showed satisfactory healing with no signs of infection or flap compromise (Figures 6, 7).

Recurrence and salvage reconstruction

**Figure 8:**
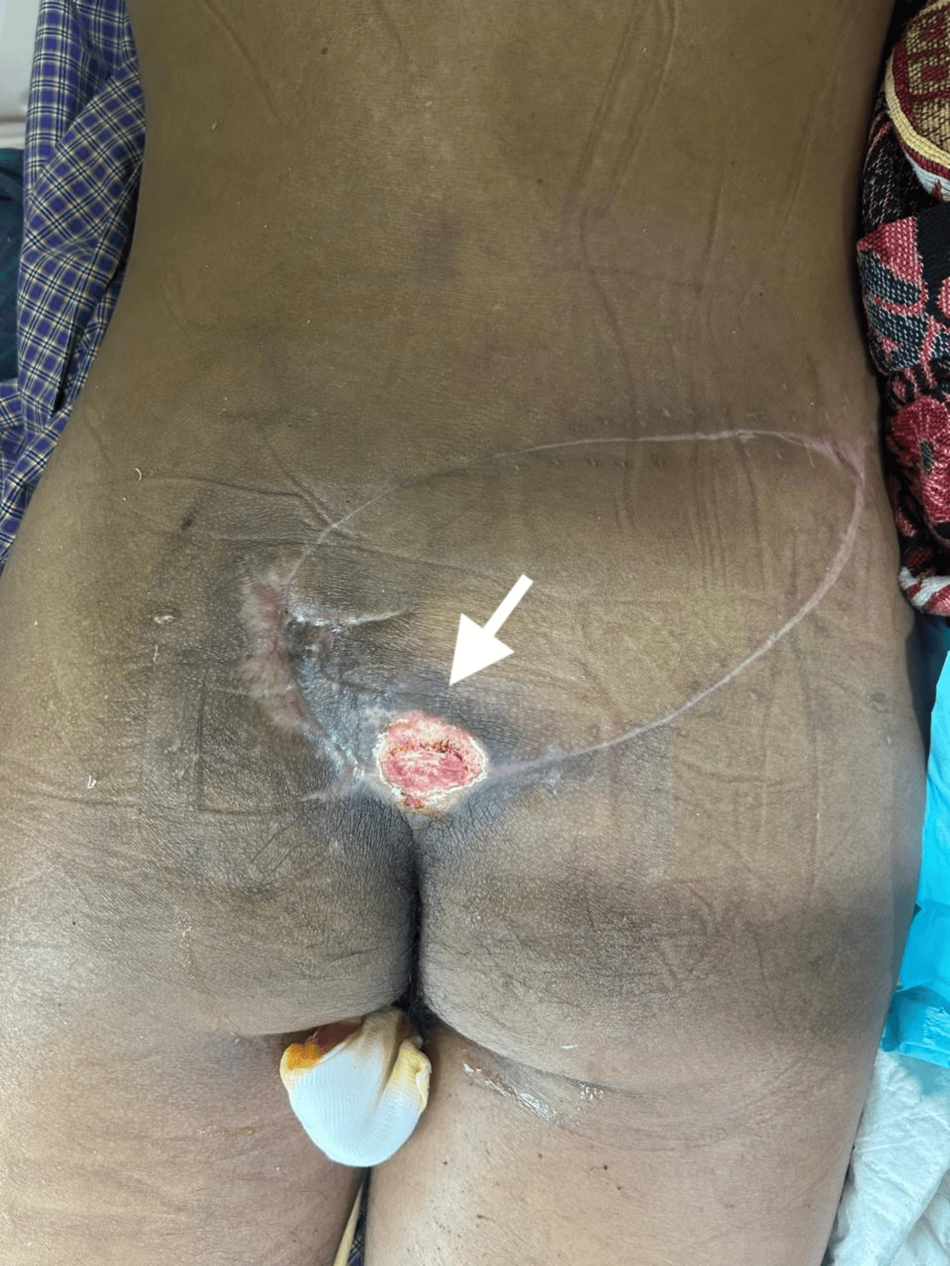
Early recurrence of sacral ulcer Recurrent ulceration at the sacral region observed four months after the initial superior gluteal artery perforator (SGAP) flap reconstruction.

Despite initial successful healing, the patient developed recurrent ulceration at the sacral region four months after surgery (Figure 8).

**Figure 9:**
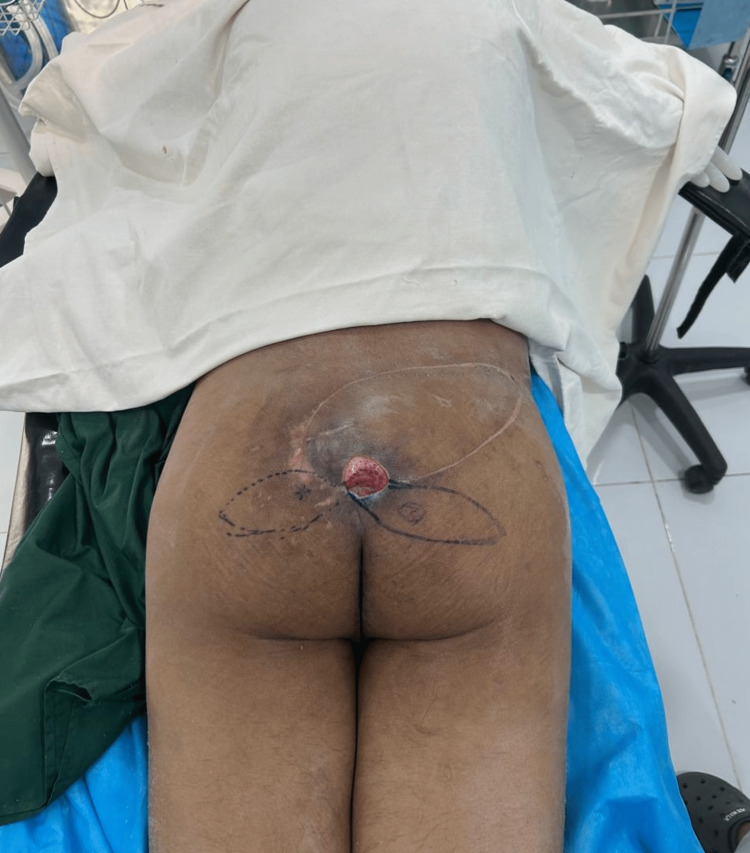
Design of the freestyle perforator flap Preoperative planning for salvage reconstruction using a freestyle perforator flap, based on perforators identified intraoperatively.

Following repeat debridement and wound preparation, reconstruction was planned using a freestyle perforator flap, based on available perforators in the surrounding tissue (Figure 9).

**Figure 10:**
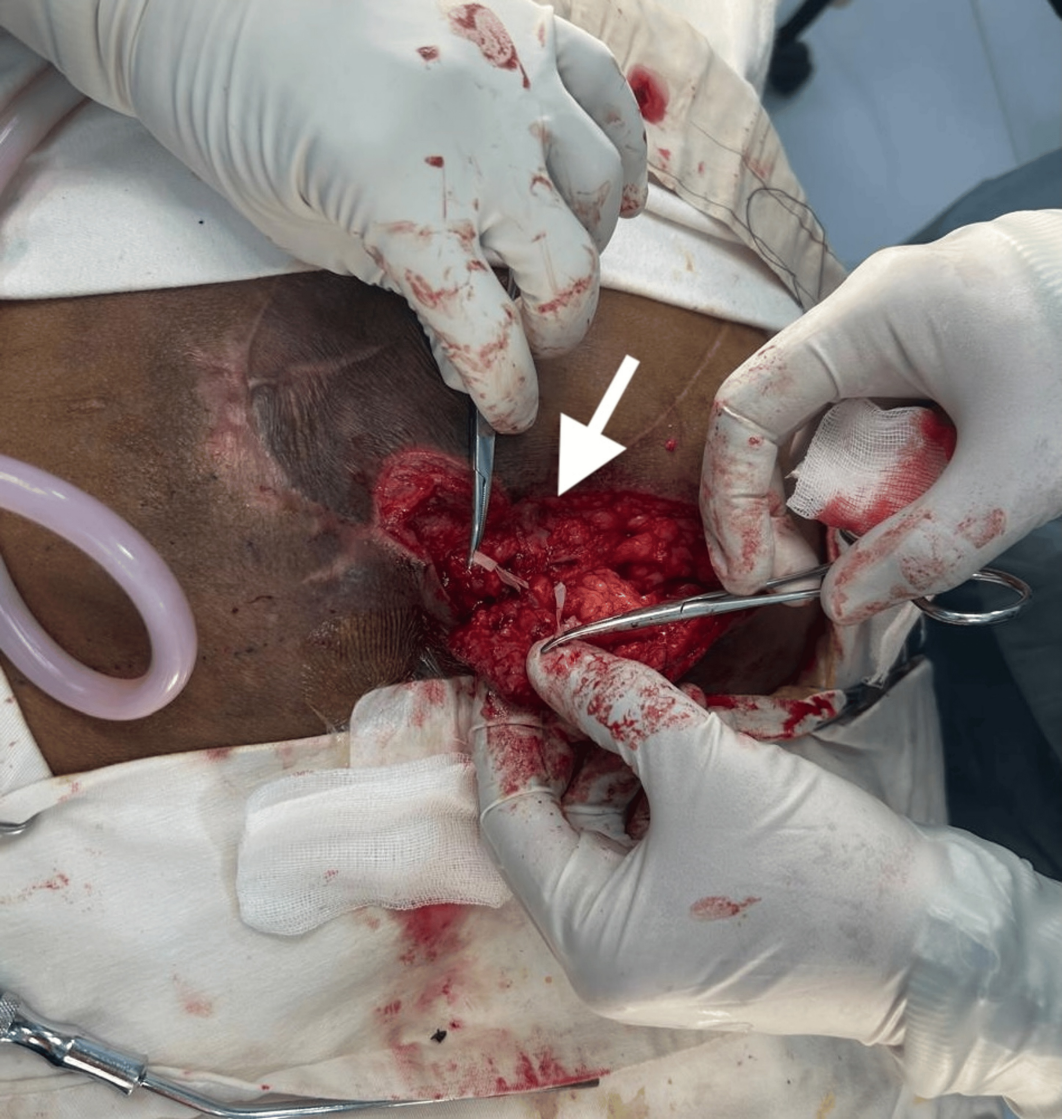
Intraoperative identification of perforator during the salvage procedure Careful dissection revealing the perforator vessel supplying the freestyle flap used for reconstruction of the recurrent ulcer (arrow).

**Figure 11:**
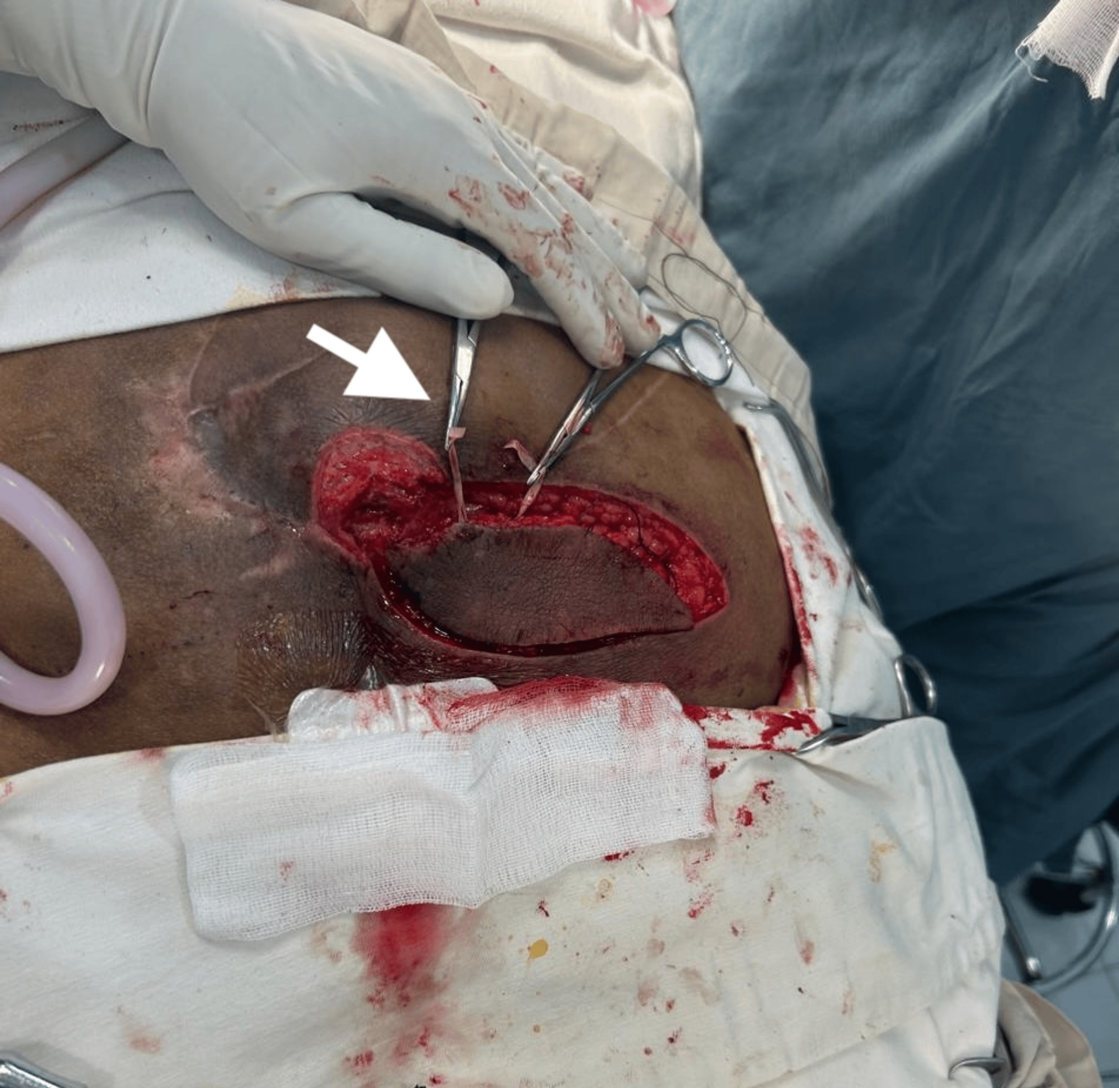
Salvage flap elevation Intraoperative photograph demonstrating elevation of the freestyle perforator flap for coverage of the recurrent sacral defect (arrow).

**Figure 12:**
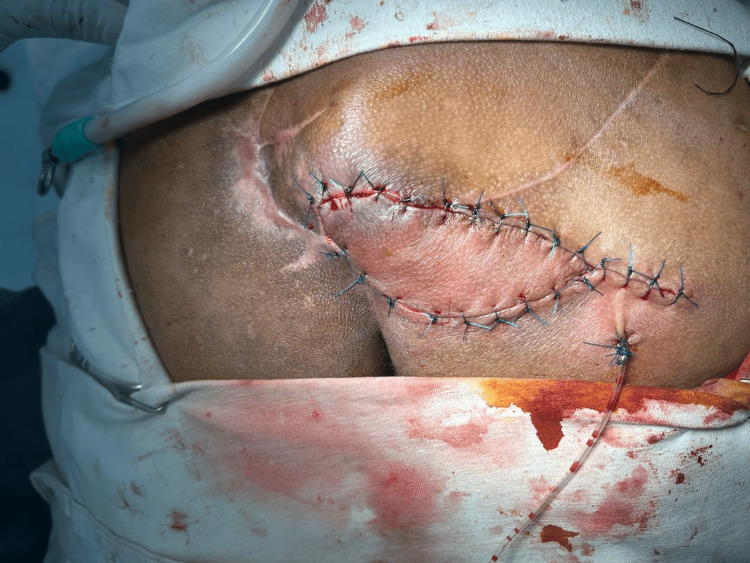
Immediate postoperative appearance after salvage reconstruction Final inset of the freestyle perforator flap with layered closure and placement of surgical drainage.

During surgery, a suitable perforator was identified intra-operatively, and a fasciocutaneous freestyle perforator flap was elevated and transposed to cover the recurrent sacral defect, and layered closure was performed with placement of a suction drain (Figures 10-12).

Postoperative off-loading protocol

**Figure 13:**
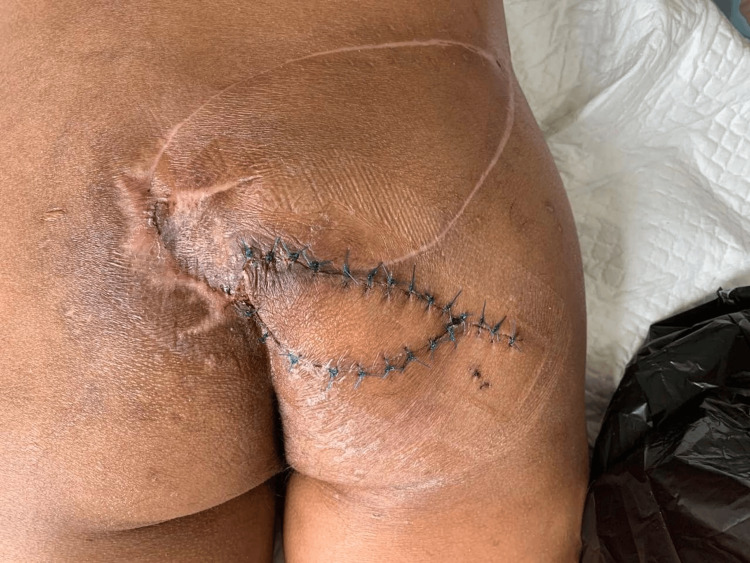
Two-week postoperative follow-up Healing flap with satisfactory tissue coverage and viable skin paddle. Sutures remain in place with no evidence of infection or necrosis.

Postoperative pressure management was strictly implemented to protect the flap and promote wound healing. During the first two weeks, the patient was maintained in prone or lateral positions with strict off-loading of the sacral region (Figure 13).

Sitting was avoided during this period. Gradual sitting was introduced after the third postoperative week, beginning with short durations of approximately 10-15 minutes twice daily. The sitting duration was progressively increased over the following weeks, reaching approximately one hour with regular pressure relief intervals. A pressure-relieving mattress was used, and the patient was educated on pressure off-loading techniques to prevent recurrence.

Follow-up

**Figure 14:**
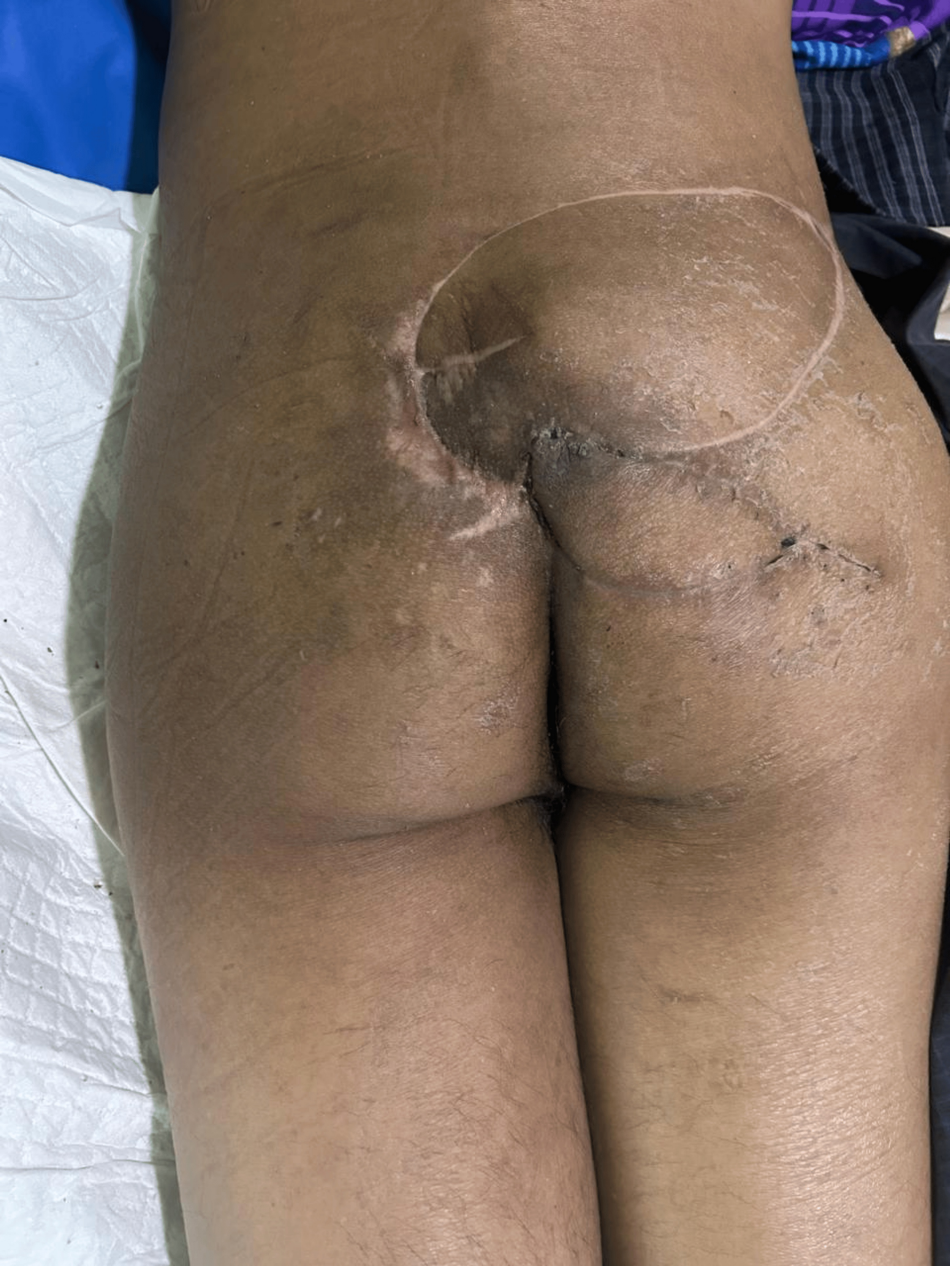
Final follow-up result Six-month postoperative follow-up demonstrating complete wound healing with stable soft tissue coverage and no recurrence.

At six months follow-up, the surgical site demonstrated complete healing with stable soft tissue coverage and no recurrence of the pressure ulcer (Figure 14).

Surgical technique

Debridement and Wound Bed Preparation

After admission, the patient underwent staged surgical debridement to remove necrotic tissue and establish a healthy wound bed. The ulcer initially measured 11 × 9 cm and demonstrated exposed sacral bone consistent with a stage IV pressure injury. Serial debridement was performed under sterile conditions. Devitalized tissue and fibrotic scar tissue were excised, and the exposed sacral bone was burred using a surgical burr to remove cortical irregularities and potential bacterial biofilm. This technique also promotes bleeding cancellous bone and improves vascularization of the wound bed. Following serial debridement, the wound size was reduced to 8 × 8 cm, with healthy granulation tissue and no signs of infection.

SGAP Flap Elevation

The patient was positioned prone. The gluteal region was prepared and draped in a sterile fashion. Perforator localization was performed through intraoperative exploration, identifying a suitable perforator arising from the superior gluteal artery. A fasciocutaneous flap, measuring 8 × 8.5 cm, was designed over the gluteal region centered over the perforator. After identification of a suitable perforator through intraoperative exploration, the skin paddle was designed according to the defect size. The incision was made along the marked flap outline, and careful dissection was performed through the subcutaneous tissue to identify and preserve the perforator vessel. The flap was elevated in a fasciocutaneous plane, while maintaining adequate perforator length and vascular integrity. Once the flap was fully mobilized, it was rotated into the sacral defect without tension. Hemostasis was secured, and the flap was inset in layers. The donor site was closed primarily with placement of a closed suction drain.

Four months after the initial reconstruction, recurrence of the sacral ulcer was observed. After repeat debridement, a freestyle perforator flap was planned and designed based on any suitable perforator identified intraoperatively rather than a predefined anatomical flap territory. A suitable perforator was identified near the defect. A fasciocutaneous flap was designed around this perforator and elevated carefully to preserve its vascular supply. The flap was then transposed into the recurrent sacral defect, and the donor site was closed primarily. A closed suction drainage was again placed. This approach allowed reconstruction of the recurrent ulcer without sacrificing additional muscle or requiring free tissue transfer.

## Discussion

Stage IV sacral pressure ulcers represent one of the most challenging reconstructive problems in patients with spinal cord injury. The combination of impaired sensation, prolonged pressure exposure, muscle atrophy, and limited mobility predisposes these patients to both ulcer formation and recurrence after reconstruction [1]. Surgical management, therefore, requires not only reliable wound coverage but also long-term preservation of reconstructive options.

Historically, gluteus maximus musculocutaneous flaps have been widely used for sacral pressure ulcer reconstruction due to their robust vascularity and ability to provide bulky tissue coverage. However, these flaps sacrifice functional muscle tissue and may compromise future reconstructive possibilities, particularly in patients with recurrent disease [3]. In recent decades, the development of perforator-based flap techniques has shifted reconstructive strategies toward muscle-sparing approaches that preserve underlying anatomy while maintaining reliable vascular supply.

The SGAP flap is widely used for sacral pressure ulcer reconstruction because it provides reliable vascularity while preserving the gluteus maximus muscle [1,8,9]. First described as a muscle-sparing alternative to the gluteus maximus musculocutaneous flap, the SGAP flap provides durable fasciocutaneous coverage with minimal donor-site morbidity while preserving the gluteal musculature [4]. Several studies have demonstrated that SGAP flaps provide reliable perfusion, adequate arc of rotation, and favorable postoperative outcomes in sacral reconstruction [5]. Additionally, preservation of the gluteus maximus muscle allows maintenance of future reconstructive options, in case of recurrence.

Despite advances in reconstructive techniques, recurrence remains a significant problem in the management of pressure ulcers. Reported recurrence rates after surgical reconstruction range from 20-40%, particularly in patients with spinal cord injury who continue to experience prolonged pressure loading and impaired sensory feedback [6,9,10]. Factors associated with recurrence include inadequate pressure off-loading, poor nutritional status, persistent mechanical stress, and local tissue compromise [6].

In the present case, the patient underwent successful primary reconstruction using an SGAP flap following thorough wound bed preparation with serial debridement and burring of exposed sacral bone. Initial postoperative healing was satisfactory. However, recurrence occurred four months later, which is consistent with the known high recurrence rates reported in the literature for spinal cord injury patients.

The occurrence of recurrence in this setting highlights the importance of selecting reconstructive techniques that preserve adjacent tissues and perforators for future salvage procedures. In our patient, the initial SGAP reconstruction preserved surrounding vascular territories, which enabled successful secondary reconstruction using a freestyle perforator flap.

Salvage reconstruction using freestyle perforator flaps have gained increasing popularity in reconstructive surgery because they enable surgeons to design flaps based on any suitable perforator identified intraoperatively rather than relying solely on predetermined anatomical territories [7,11,12]. This technique offers several advantages, including flexibility in flap design, preservation of muscle tissue, and the ability to reconstruct defects in previously operated regions.

Tissue-preserving reconstructive strategies are essential for patients with high recurrence risk because preservation of perforators allows future reconstructive options if there is recurrence [12,13]. Because the flap is designed around a perforator identified intraoperatively, surgeons can adapt the flap orientation and dimensions to match the specific characteristics of the defect.

In this case, the freestyle perforator flap allowed effective salvage reconstruction of the recurrent sacral ulcer without sacrificing additional muscle or requiring free tissue transfer. This approach minimized donor site morbidity while achieving durable wound coverage.

Another critical component of successful pressure ulcer reconstruction is postoperative management. Strict pressure off-loading protocols, gradual reintroduction of sitting, and specialized pressure-relieving mattresses are essential to prevent recurrence. Education of both patients and caregivers regarding pressure relief strategies plays a vital role in long-term outcomes.

This case highlights several important principles in the surgical management of sacral pressure ulcers. Thorough wound bed preparation, including serial debridement and removal of nonviable bone, is essential before definitive reconstruction. Perforator-based flaps such as the SGAP flap provide reliable coverage while preserving muscle and future reconstructive options. Freestyle perforator flaps represent a valuable salvage option for recurrent pressure ulcers in previously reconstructed areas. In addition, strict postoperative pressure management and rehabilitation protocols remain essential in preventing recurrence.

By employing a sequential perforator-based reconstructive strategy, successful healing was achieved despite recurrence, demonstrating the versatility and durability of perforator flap techniques in complex sacral pressure ulcer reconstruction.

Clinical message

**Figure 15:**
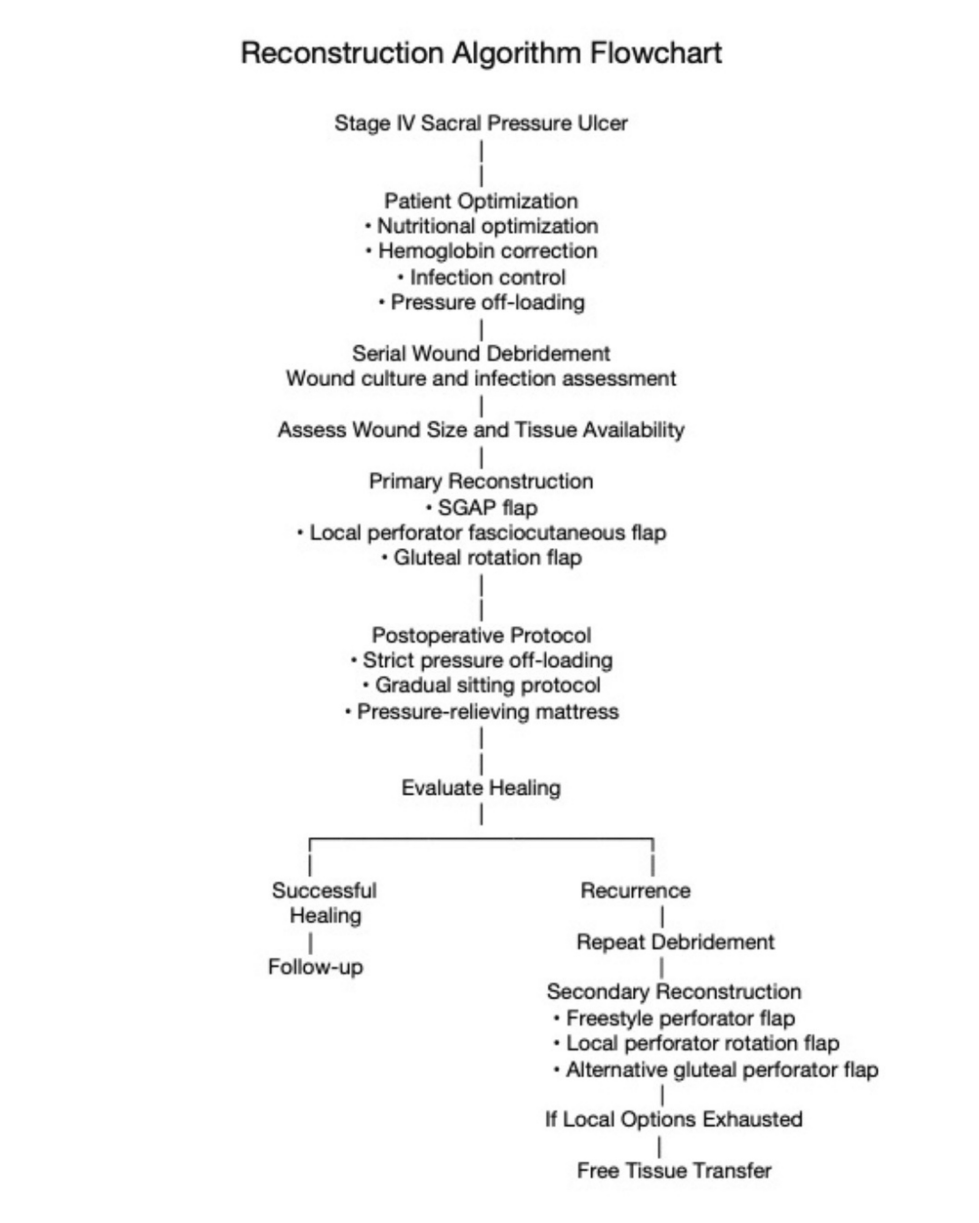
Proposed algorithm for reconstruction of stage IV sacral pressure ulcers This algorithm illustrates a reconstructive decision-making pathway for the management of stage IV sacral pressure ulcers in patients with spinal cord injury. Initial management involves patient optimization, infection control, and serial wound debridement. Definitive reconstruction may be performed using perforator-based flaps such as the superior gluteal artery perforator (SGAP) flap. In cases of ulcer recurrence, tissue-preserving reconstruction allows secondary procedures such as freestyle perforator flaps or other local perforator flaps. Free tissue transfer may be considered when local reconstructive options are exhausted. The algorithm is based on established reconstructive principles described in previous studies [1,3-7]. Image credit: Created by Dr. Zin using BioRender.com (Toronto, Canada; no generative AI tools used).

Based on our experience and previously reported reconstructive strategies, we propose an algorithm for the surgical management of stage IV sacral pressure ulcers in patients with spinal cord injury (Figure 15).

## Conclusions

Stage IV sacral pressure ulcers in patients with spinal cord injury remain challenging due to high recurrence rates and persistent risk factors such as prolonged pressure exposure and impaired sensation. Perforator-based flap reconstruction offers reliable soft tissue coverage while preserving underlying musculature and future reconstructive options.

In this case, primary reconstruction using an SGAP flap provided effective initial coverage. When recurrence occurred, preservation of regional vascular territories allowed successful salvage reconstruction using a freestyle perforator flap, resulting in complete wound healing at six-month follow-up.

This case highlights the importance of tissue-preserving reconstructive strategies and careful long-term planning in the management of pressure ulcers in spinal cord injury patients. Sequential use of perforator-based flaps may provide an effective approach for managing recurrent sacral pressure ulcers while maintaining future reconstructive options
